# Measurement and Analysis of P2P IPTV Program Resource

**DOI:** 10.1155/2014/101702

**Published:** 2014-03-19

**Authors:** Wenxian Wang, Xingshu Chen, Haizhou Wang, Qi Zhang, Cheng Wang

**Affiliations:** ^1^Network and Trusted Computing Institute, College of Computer Science, Sichuan University, Chengdu 610065, China; ^2^Mathematical College, Sichuan University, Chengdu 610064, China

## Abstract

With the rapid development of P2P technology, P2P IPTV applications have received more and more attention. And program resource distribution is very important to P2P IPTV applications. In order to collect IPTV program resources, a distributed multi-protocol crawler is proposed. And the crawler has collected more than 13 million pieces of information of IPTV programs from 2009 to 2012. In addition, the distribution of IPTV programs is independent and incompact, resulting in chaos of program names, which obstructs searching and organizing programs. Thus, we focus on characteristic analysis of program resources, including the distributions of length of program names, the entropy of the character types, and hierarchy depth of programs. These analyses reveal the disorderly naming conventions of P2P IPTV programs. The analysis results can help to purify and extract useful information from chaotic names for better retrieval and accelerate automatic sorting of program and establishment of IPTV repository. In order to represent popularity of programs and to predict user behavior and popularity of hot programs over a period, we also put forward an analytical model of hot programs.

## 1. Introduction

Peer-to-Peer (P2P) applications take advantage of resources such as storage, CPU cycles, content, or human presence available at the edge of the Internet to provide a service [1]. With the development and maturity of P2P technology, P2P applications become more and more popular in the recent ten years, including file-sharing applications, audio-based VOIP applications, and video-based IPTV applications [2–5]. However, they occupy a significant proportion of Internet traffic. According to a survey from CacheLogic [6] in June, 2004, 60% of the Internet traffic is P2P. In addition, P2P IPTV applications, such as PPTV (former PPLive) [2], QQLive [4] in China, become popular gradually and occupy a great amount of P2P traffic [7].

P2P IPTV, also called P2P streaming, emerged recently as a novel framework to deliver TV programs or live events to a large number of viewers over the Internet. With the rapid large-scale popularization of broadband technology, P2P IPTV becomes the disruptive IP communication technology, which greatly revolutionizes people's lives and entertainment [8]. Several P2P IPTV applications have gained great commercial success, including CoolStreaming [9], PPTV, PPStream, and UUSee. With low price and simple operation, P2P IPTV becomes more popular in recent years and receives great attention from both industry and academia. It was reported that PPTV had more than 200 million installations and its active monthly user base (in December 2010) was 104 million. PPTV had the penetration of 43% in Chinese Internet users [10].

There are more and more P2P IPTV applications in the Internet now. And it is difficult to measure the applications because they use proprietary protocols. Source codes or official documents are scarcely published. However, the measurement of P2P IPTV applications is an important problem for the management and development of IPTV and several researchers have tried to address this issue. But none of them focused on program-list and its distribution of P2P IPTV. In this paper, we proposed a distributed multiprotocol crawler (DMP-Crawler) for collecting program resources in P2P IPTV networks. Moreover, we analyzed the characteristics of these IPTV programs and presented an analytical model of hot programs. The model can be used to infer the popular drama IPTV users at some time.

The remainder of this paper is structured as follows.  Section 2 presents an overview of P2P IPTV.  Section 3 introduces related work of P2P IPTV measurement.  Section 4 describes the principle of P2P IPTV program-list distribution, DMP-Crawler, and methodology of measurement and analysis.  Section 5 presents and discusses the results. Finally, Section 6 concludes the paper and gives the future work.

## 2. Overview of P2P IPTV

### 2.1. P2P IPTV

Internet Protocol Television (IPTV) denotes the transport of live streams and recorded movies or video clips by means of advanced packet-switched Internet technologies [11]. ITU-T defined IPTV as multimedia services such as television/video/audio/text/graphics/data delivered over IP-based networks managed to support the required level of QoS/QoE/, security, interactivity, and reliability [12].

Over the past decade, P2P technology has been a promising solution for the distribution of large-scale media and a large amount of P2P IPTV systems have been developed and widely deployed on the Internet. In this paper, we defined P2P IPTV as a technology that enabled users to transmit and receive multimedia services including television, video, audio, text, and graphic through P2P overlay networks with support for QoS/QoE, security, mobility, interactivity, and reliability. Through P2P IPTV, users can enjoy IPTV services anywhere. Now P2P IPTV applications are changing the way that we watch TV and movies.

In 2000, Chu et al. proposed End System Multicast (ESM) [13], the first P2P IPTV application, in which an overlay tree is constructed to distribute video data and continuously optimized to minimize end-to-end latency. Then the overlay networks are adopted for efficient distribution of live video. The overlay networks include Nice [14], SplitStream [15], Scattercast [16], and Overcast [17]. Unfortunately, they were not deployed in large scale due to their limited capabilities. CoolStreaming was released in summer 2004 and arguably represented the first large-scale P2P video streaming experiment [9]. Then, CoolStreaming was significantly modified and commercially launched. With the success of CoolStreaming, many P2P IPTV applications emerged in 2005. The known applications include PPLive, PPStream, QQLive, and UUSee in China. From 2006, related measurements of P2P IPTV were done by a number of academic staff, and we also carried out the measurement work [18] in 2007.

### 2.2. Architecture of P2P IPTV

**Figure 1:**
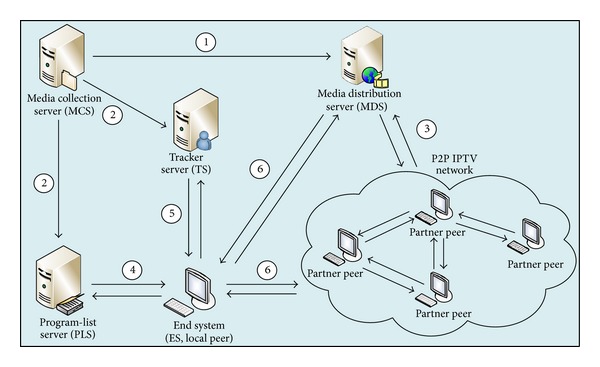
Architecture of P2P IPTV.

A typical P2P IPTV application is comprised of five components: media collection server (MCS), media distribution server (MDS), program-list server (PLS), tracker server (TS, also called peer-list server), and end system (ES, also called client or peer). As illustrated in Figure 1, the basic workflow of a P2P IPTV application is provided as follows.

**Step 1**

MCS gathers video data in two ways. Firstly, for live program, MCS gets video data from video grabber. Secondly, for video on demand (VoD), MCS reads video file directly, encodes video data according to some coding methods, and uploads the data to MDS.

**Step 2**

When coding data of a video, MCS will generate the related program name, program GUID (Globally Unique Identifier), play link, category, and so forth and register the information in PLS. At the same time, MDS will register program GUID in TS.

**Step 3**

After receiving live data, MDS will distribute them to IPTV network. After receiving VoD data, MDS will store them firstly and distribute them when clients request them. We have introduced the video distribution protocol in detail in 2012 [18].

**Step 4**

The local peer requests the latest program-list file from PLS and updates it immediately after lunching the IPTV client. The list of program consists of program name, program GUID which is the most important identification of signal communication among peers, program descriptions, and so forth.

**Step 5**

After the local peer selects one program to watch videos, the peer registers itself to the tracker server and sends multiple query messages to the server to retrieve a small set of partner peers who are watching the same program. The information of partner peers includes IP address, TCP port, and UDP port. Upon receiving the initial list of partner peers, the local peer uses this seed peer list to harvest additional lists by periodically probing active peers which maintain a list of peers.

**Step 6**

After harvesting enough peers, the peer tries to connect the active ones or MDS to request video data for playback of the appointed program and launches a local media player (such as Windows Media Player and RealPlayer) to play the video. To deal with the churn of peers, the local peer needs to actively seek new peers from its existing partners to update peer list. At the same time, it also rebroadcasts its current peer list to its partner peers.

Our work is focused on Step 4, such as the distribution of program-list.

## 3. Related Work

P2P IPTV measurement has been extensively studied. The measuring methods can be classified into two types: passive tracing and active tracing approach.

The passive approach is performed by deploying code at suitable points in the network infrastructure. The passive approach does not increase the network traffic. And it is often used to analyze and identify P2P IPTV traffic from general Internet traffic with the known behaviors (such as connection ports, feature, or patterns). It is also used to capture IPTV traces and grasp the P2P IPTV application. Du et al. [19] and Tan et al. [20] developed a machine learning methodology to identify PPLive and PPStreasm traffic. Agarwal et al. [21] studied the program startup time and the quality of service in terms of the number of consecutive lost block. Silverston and Fourmaux [22] studied four IPTV applications and gave the global view of the impact of P2P media streaming on the network traffic. Following the research, they presented a detailed study of IPTV traffic, providing useful insights on transport-level and packet-level properties as well as the behaviors of the peers in the network [23]. With abundant traces from a successful commercial P2P IPTV application, Wu et al. [24] characterized interpeer bandwidth availability in large-scale P2P streaming networks. The passive approach is potentially transparent and scalable and allows the comparison of traffic from multiple domains side-by-side. However, it is dependent upon the access to core network infrastructure, which is not always available. Thus, it is often used for flow control in firewall or gateway devices.

In the active approach, the special crawler, like an ordinary client, is adopted to inject test packets into P2P IPTV network or send packets to servers and peers. Then the crawler follows packets and measures characters of IPTV network. Hei et al. [25] carried out the first active tracing of a commercial P2P IPTV application, namely, PPLive. They further developed a dedicated PPLive crawler to study the global characteristics of PPLive system [26]. Wu et al. [27] presented Magellan to characterize topologies of peer-to-peer streaming networks of UUSee. Vu et al. [28] mapped the PPLive network to study the impacts of media streaming on P2P overlays.

Most of existing research work surveyed the P2P IPTV network-centric metrics (such as traffic characterization, TCP or UDP connections, and video traffic) or user-centric metrics (such as user arrival and departure, geographic distribution, and channel population). Our studies were primarily focused on program-list distribution of P2P IPTV applications because program resource distribution was very important to P2P IPTV applications. Our work surveyed the P2P IPTV content-centric metrics which were useful for prediction and monitoring of programs. In this paper, a distributed multiprotocol program crawler was proposed to collect various kinds of information of programs. Moreover, we also analyzed the characteristics of program resources and put forward an analytical model of hot program.

## 4. Methodology of Measurement and Characteristic Analysis

In this section, we will present the basic principle of program-list distribution in P2P IPTV applications and illustrate a feasible and efficient architecture for crawling program-list.

### 4.1. Principle of Program Resource Distribution

When the program-list is downloaded and extracted by an IPTV client, users can select a program to watch videos. So program-list distribution is very important in P2P IPTV applications. The program-list includes program name, categories, play-link, and descriptions. Play-link is the most important identification of signal communication among peers viewing the same program. A typical example of program metadata is shown in Table 1.

The client-server architecture, as shown in Figure 1, is usually used to distribute program-list file in IPTV systems. When an IPTV client starts up, it requests program-list file from program-list servers and updates the local information of all the programs immediately. XML is usually used in program-list files to organize various metadata of programs. This is different from website-based program-list distribution of video-sharing sites like Youku and YouTube. Program-list of IPTV is well organized for convenient browsing.

With the rapid increase in the number of programs, the size of program-list file becomes bigger and bigger. For example, PPTV had about 300 thousand programs in 2011, and the size of program-list file was more than 20 MB, which is a heavy burden to program-list servers and leads to bad experience to users. Some IPTV applications use compression method to decrease the file size, while others use multiple program-list files based on program categories. Furthermore, some IPTV applications encrypt program-list files to prevent hotlinking.

### 4.2. Architecture of DMP-Crawler

In order to obtain program information of IPTV applications, it is necessary to summarize the principle of program-list distribution of the most of IPTV applications and decrypt the encrypt algorithm and XML metadata of program-list file.

**Figure 2:**
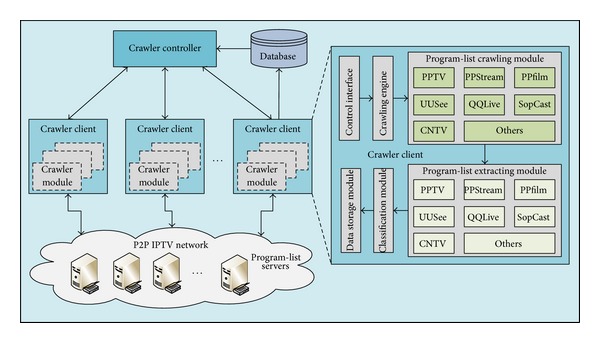
Architecture of DMP-Crawler.

Then, an efficient distributed multiprotocol crawler (DMP-Crawler) was proposed to collect various kinds of information of programs in popular P2P IPTV applications. {Program name, IPTV application name} was used to uniquely identify a program.  Figure 2 presents an overview of architecture of DMP-Crawler, which is composed of one crawler controller and a number of crawler clients.

On the basis of crawler clients' and server's status, the crawler controller assigns tasks to multiple independent crawler clients through a task scheduling algorithm. Each crawler client periodically reports its crawling status as well as CPU and memory consumption to crawler controller.

A crawler client mainly includes crawling engine, program-list crawling module, program-list extracting module, classification module, and data storage module. According to crawling task type, the crawler client invokes crawler engine, requests program-list file from program-list servers, and reports crawling status to crawler controller. When program-list file is downloaded, the crawler client extracts metadata of programs from the file, classifies these programs, and stores all information into database for further analyses.

### 4.3. Characteristic Analysis of Program Resource

In order to understand naming rules of IPTV programs, characteristics of program naming were analyzed with statistical methods. Characteristics analysis of programs included distributions of the length of program names, the entropy of the character types, high-frequency symbols in the names, and distributions of the hierarchy depth of program names.

**Definition 1**

Program Name (PN) is composed of a serial of characters, PN = *c*_1_ *c*_2_ ⋯ *c*_*n*_, Where *n* is the number of characters or length of PN and *n* = len(PN). *c*_*i*_  (*i* = 1, 2, …, *n*) is a printable character in some coded character set, such as Chinese, English, Latin, and punctuation symbol.

 If len(PN) ≤ 10, the program has a short name.
 If 10 < len(PN) ≤ 20, the program has a medium name.
 If 20 < len(PN) ≤ 30, the program has a long name.
 If len(PN) > 30, the program has a super-long name.

Let a random variable *x* denote the character type of program name. The set of value of *x* is denoted as

(1) $$\begin{array}{c} \text{CharsType}=\left\{\text{C},\text{E},\text{L},\text{G},\text{N},\text{S},\text{O}\right\}, \end{array}$$

where C, E, L, G, N, S, and O represent Chinese, English, Latin, Greek, Number, Symbol (includes punctuation and special character), and unidentified character, respectively. Character type is defined by Unicode Character Database (UCD) [29].

Let *U*_*c*_ denote the set of characters of program names, *c*_*i*_ ∈ *U*_*c*_.

With a mapping function *f* : *U*_*c*_ → CharsType, every character of program name can be transformed to the corresponding character type as follows:

(2) $$\begin{array}{c} f\left(\text{PN}\right)=x_{1}x_{2}\cdots x_{n}, \end{array}$$

where *x*_*i*_ ∈ CharsType, *i* = 1, 2, …, *n*.

Let *H*(CharsType) denote the information entropy of *x*_*i*_, which is used to evaluate the chaos of program naming [30]

(3) $$\begin{array}{c} H\left(\text{CharsType}\right)=-\overset{V}{\underset{i=1}{\sum }}p\left(x_{i}\right)\cdot \log_{2}\left(p\left(x_{i}\right)\right), \end{array}$$

where *p*(*x*_*i*_) is the probability of *x*_*i*_; *V* is the number of character type in program name. Thus, the value of *H*(CharsType) is between 0 and log_2_ *V*. In the calculation of entropy, let 0 · log_2_0 = 0.

### 4.4. Analytical Model of Hot Programs

**Definition 2**

Hot programs are the top 100 popular programs that have the most viewers and concern a large amount of people.

**Definition 3**

Hot degree is used to describe the concerned degree or level of hot programs by people. The influencing factors of hot degree include the number, watching time, and comments of viewers.

Let HD denote the hot degree of a program. In a P2P IPTV application, it can be expressed as

(4) $$\begin{array}{c} \text{HD}=\alpha \cdot U+\beta \cdot T+\gamma \cdot C,\\ \alpha +\beta +\gamma =1, \end{array}$$

where *U*, *T*, and *C* represent the number of viewers, watching time, and the number of comments, respectively. Here the number of viewers refers to the number of online viewers at some point. And *α*, *β*, and *γ* are their weights.

For all P2P IPTV applications, (4) can be rewritten as

(5) $$\begin{array}{c} \text{HD}=\overset{j}{\underset{i=1}{\sum }}\text{H}{\text{D}}_{i}=\overset{j}{\underset{i=1}{\sum }}\left(\alpha \cdot U_{i}+\beta \cdot T_{i}+\gamma \cdot C_{i}\right),\\ \alpha +\beta +\gamma =1, \end{array}$$

where *j* is the number of IPTV applications.

## 5. Results and Discussion

### 5.1. Crawling Results of DMP-Crawler

DMP-Crawler consists of one crawler controller and ten crawler clients. DMP-Crawler is deployed on three PC Servers with Intel E5506 CPU and 4 GB Memory in Beijing of China with 10 Mbps Ethernet network access. DMP-Crawler ran two rounds and collected about 900,000 programs every day. According to the collected program names and IPTV application names, the repeated programs were removed.

From February 2009 to July 2012, DMP-Crawler collected 13,107,766 distinct programs from 33 IPTV applications in China, in which only 0.3% of the programs were live programs. In particular, PPfilm has no live progr am.

**Figure 3:**
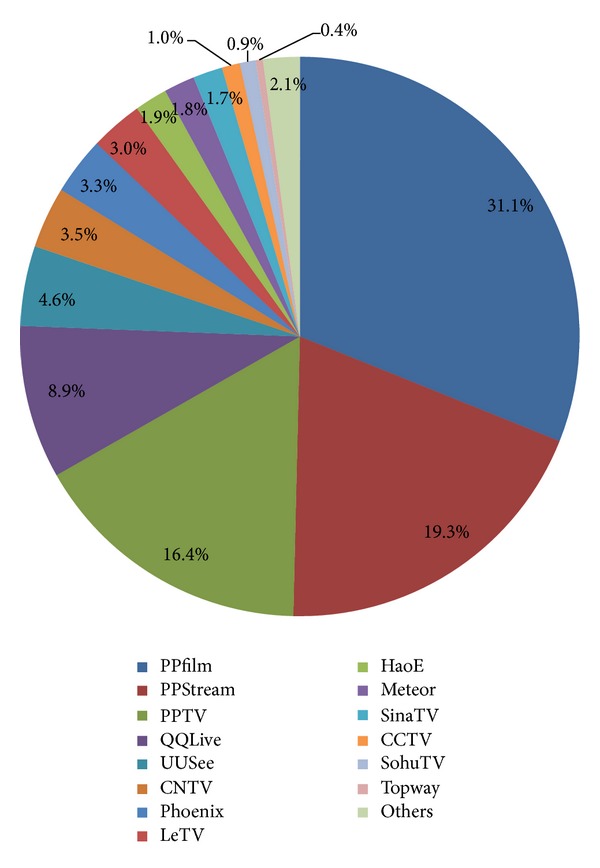
Distribution of various IPTV programs.

The numbers and ratios of programs of 33 IPTV applications are shown graphically in Figure 3. From the collected data, we can find that the distribution of programs is highly skewed. The most popular IPTV application is PPfilm, accounting for about 31.1%, and the second is PPStream, accounting for about 19.3%. These two IPTV applications account for about one half of the entire IPTV programs.

According to the requirements of the State Administration of Radio Film and Television (SARFT), IPTV service providers must apply for “Information Network Dissemination Audio-Visual Programs Permit” before August 2009. Some IPTV service providers could not acquire the permit and stopped video service in 2010. Therefore, these IPTV applications have only hundreds or thousands of programs.

**Figure 4:**
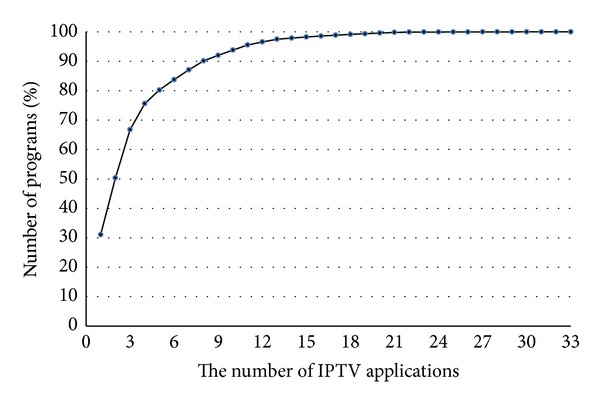
CDF of distribution of programs.

We ranked each of the IPTV applications according to their percentages of programs and plotted a typical Cumulative Distribution Function (CDF) of the percentages of programs in Figure 4.

In Figure 4, 15.2% (5/33) popular IPTV applications have 80% programs and 24.2% (8/33) popular IPTV applications have more than 90% programs. Some IPTV applications, like SopCast and TVUPlayer, have only a small proportion of programs, for they have no video on demand.

**Figure 5:**
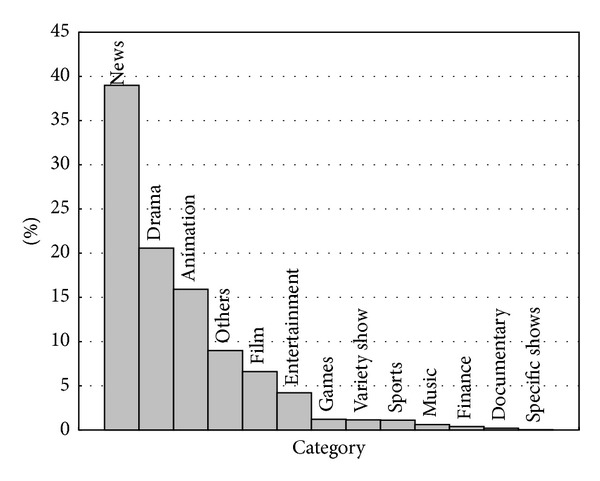
Distribution of programs' categories.

When programs were extracted from program-list file, programs were classified into one of 13 categories defined by SARFT. Percentages of all the categories are shown graphically in Figure 5. From the figure, we can observe that the distribution is highly skewed: the most popular category is News, accounting for about 39%; the second is Drama, accounting for about 21%; the third is Animation, accounting for about 16%; and the last is Specific show, accounting for about 0.02%.

In Figure 5, we also list category “Others.” “Others” are programs that cannot be classified.

### 5.2. Characteristic Analysis of Program Resource

#### 5.2.1. Distribution of Length of Program Name

All of the programs were sorted according to the length of name and percentages of programs were calculated according to the length interval of 5. Percentages of all the length intervals are shown in Table 2.

In Table 2, about 40% of programs have short names; 48.5% of programs have medium names; 8.8% of programs have long names; only 3.5% of programs have super-long names. In the 5 popular IPTV applications, more than 30% of PPTV programs of PPTV application have long and superlong names. 75.2% of QQLive and 58.8% of PPfilm programs have short names.

We also analyze quartile of name length. The results are shown in Table 3. QQLive programs have the smallest *Q*_1_ and interquartile range, and PPTV programs have the biggest *Q*_3_ and interquartile range, which are in accordance with the results in Table 2.

From Tables 2 and 3, we can infer that short and medium names are often used in IPTV program naming, especially in QQLive programs.

#### 5.2.2. Character Type of Program Name

All characters in program name were counted and mapped to corresponding CharsType. And the character types include C, E, L, G, N, S, and O. The number of character types is 7. Probabilities of C, E, L, G, N, S, and O are 0.585135, 0.051970, 0.00950, 0.000029, 0.199762, 0.153493, and 0.000109, respectively. The chaos of IPTV program naming is 1.122197 according to (3).

When collecting IPTV programs, we also put forward a BitTorrent crawler and an eDonkey crawler to crawl program resources in BitTorrent and eDonkey network. The two crawlers collected 2,329,237 BitTorrent programs and 619,810 eDonkey programs.

In Table 4, information entropy of character of IPTV programs is less than that of BitTorrent and eDonkey programs. The chaos of IPTV program naming is small, indicating that IPTV program naming is relatively regular. It may be interpreted as follows: popular P2P IPTV applications are operated commercially, while BitTorrent and eDonkey are public platforms and the programs are uploaded by amateurs.

#### 5.2.3. Hierarchy Depth of Program

**Definition 4**

Hierarchy depth of program is the times that a program is classified according to category, channel, subchannel, and so forth. For example, hierarchy depth of the program in Table 1 is 2.

Original intention of statistics of hierarchy depth is to find the relationship between length and hierarchy depth. Statistical results of hierarchy depth of all programs and popular IPTV programs are presented in Table 5.

In Table 5, more than 50% programs' hierarchy depth is 2 and 27.42% programs' hierarchy depth is 3. The 2-hierarchy is easy to show programs in IPTV client. The 3-hierarchy is often used to display movies and drama programs. Hierarchy depth distributions of PPTV and PPfilm are similar. And the 2-hierarchy and 3-hierarchy programs in PPTV and PPfilm account for more than 90%. While hierarchy depth distribution of QQLive is quite different from that of other applications, its 4-hierarchy programs account for 57.22%. Thus, its programs are prone to used short name.

### 5.3. Analysis of Hot Programs

In the measurement of P2P IPTV, it is difficult to measure watching time of programs which is managed by IPTV operators. It is impossible for us to collaborate with major IPTV operators. In addition, only a few popular IPTV applications provide program comment functions in IPTV client or website. Moreover, the number of online viewers is much more than the number of comments. Here let *α* = 1, *β* = *γ* = 0; (5) was simplified as

(6) $$\begin{array}{c} \text{HD}=\overset{j}{\underset{i=1}{\sum }}\text{H}{\text{D}}_{i}=\overset{j}{\underset{i=1}{\sum }}U_{i}. \end{array}$$

From (6), we can find that hot programs appear in popular IPTV applications. Thus, we only consider the top 5 IPTV applications; then

(7) $$\begin{array}{c} \text{HD}=U_{\text{PPStream}}+U_{\text{PPfilm}}+U_{\text{UUSee}}+U_{\text{PPTV}}+U_{\text{QQLive}}. \end{array}$$

For a program, PPStream and PPfilm provide the number of its viewers, UUSee presents the ratio of its viewers, while PPTV and QQLive only offer 6-level popularity. Thus, we must normalize the number of viewers according to the number of online viewers of various IPTV applications. In June 2010, the maximum viewers of PPStream, UUSee, PPTV, and QQLive are about 20.0, 2.0, 11.0, and 6.6 million, respectively. The normalizing rules are as follows.

(1) *U*_PPStream_ and *U*_PPfilm_ can be obtained from their program-list files.
(2) UUSee presents the ratio of viewers. *U*_UUSee_ is calculated according to the ratio and the number of online viewers. We estimate the online viewers of UUSee as 2 million.
(3) PPTV and QQLive offer 6-level popularity of programs. Every popularity level presents an interval of the number of viewers. Popularity levels of 0, 1, 2, 3, 4, and 5, respectively, represent [0, 199], [200, 399], [400,599], [600, 799], [800,999], and [1000, + *∞*]. If popularity level is between 0 and 4, median of corresponding interval is used as *U*. If popularity level is 5 and PPStream has not the same program, *U*_PPTV_ = 3000 and *U*_QQLive_ = 1800. If popularity level is 5 and PPStream has the same program, *U*_PPTV_ and *U*_QQLive_ are calculated by
  (8) $$\begin{array}{c} U_{\text{PPTV}}=0.55\times U_{\text{PPStream}},\\ U_{\text{QQLive}}=0.33\times U_{\text{PPStream}}. \end{array}$$

Table 6 lists the hottest programs in one week through analytical model of hot programs. From Table 6, we can infer that the most popular drama IPTV was* Let's see the Meteor Shower together *in that week. Moreover, the model can be used to predict popularity of hot programs in some time.

## 6. Conclusions

In this paper, we have studied the program information collection in P2P IPTV applications. We proposed a distributed multiprotocol crawler to harvest program information of P2P IPTV applications. As far as we know, it is the first time that the detailed crawler for IPTV programs is presented. Characteristic analysis of programs was carried out. The results reveal the disorderly naming conventions of P2P IPTV program and can help to purify and extract useful information from chaos names for better retrieval. We also put forward an analytical model of hot programs to represent popularity of programs and predict user behaviors and popularity of hot programs within a period.

Distribution of IPTV programs is independent and incompact, resulting in the chaos of program name, which obstructs searching and organizing programs. In the next work, we will focus on data mining of programs and establishment of IPTV repository.

## Figures and Tables

**Table 1:** Metadata of a IPTV program.

Program name	110205- Happy camp
Category	Variety show
Channel name	Happy camp
Subchannel name	—
IPTV application name	PPStream
Play-link	pps://n26aeygqeb6s4kbc2aqa.pps/ 110205- Happy Camp.wmv
Number of views	425
Date added	2011/02/05

**Table 2:** Distribution of length of program name.

Length Range	PPStream	PPTV	QQLive	UUSee	PPfilm	All
(0, 5)	5.2%	3.0%	15.8%	2.8%	8.3%	9.3%
(5, 10)	44.0%	16.9%	59.4%	17.9%	50.5%	29.9%
(10, 15)	26.7%	30.2%	17.5%	26.8%	29.7%	25.1%
(15, 20)	17.6%	18.5%	5.2%	46.4%	7.3%	23.4%
(20, 25)	5.6%	12.6%	1.6%	5.1%	1.4%	6.3%
(25, 30)	0.6%	7.4%	0.3%	0.8%	0.4%	2.5%
(30, +∞)	0.2%	11.5%	0.2%	0.2%	2.5%	3.5%

**Table 3:** Length quartile of length of program name.

IPTV Applications	*Q*_1_	Median	*Q*_3_	Interquartile Range (*Q*_3_–*Q*_1_)
PPStream	8	11	15	7
PPTV	11	15	23	12
QQLive	6	7	10	4
UUSee	12	16	18	6
PPfilm	7	9	13	6
All	8	13	17	9

**Table 4:** Information entropy and probability of character type.

Character Type	Probability of character type		
	IPTV	BitTorrent	eDonkey
Chinese (C)	0.585135	0.260022	0.220748
English (E)	0.051970	0.345382	0.472992
Latin (L)	0.00950	0.002303	0.002324
Greek (G)	0.000029	0.00001	0.000013
Number (N)	0.199762	0.12577	0.057365
Symbol (S)	0.153493	0.264594	0.242756
Unidentified character (O)	0.000109	0.00194	0.003801
Information entropy (H)	1.122197	1.356355	1.231227

**Table 5:** Distribution of hierarchy depth of programs.

Hierarchy Depth	PPStream	PPTV	QQLive	PPfilm	UUSee	All
1	19.12%	3.20%	15.95%	8.34%	3.25%	14.72%
2	80.87%	44.69%	9.34%	43.35%	85.64%	50.76%
3	0.014%	52.11%	17.50%	48.31%	11.11%	27.42%
4	0.00%	0.004%	57.22%	0.003%	0.00%	7.09%
5	0.00%	0.00%	0.0003%	0.00%	0.00%	0.002%

**Table 6:** Hottest programs in one week.

Date	Program names	Categories	HD
2010-12-23	*Let's see the Meteor Shower together—23 *	Drama	116099
2010-12-24	*Salt (Angelina Jolie) *	Film	66071
2010-12-25	*100824- Kangxi *	Variety Show	94976
2010-12-26	*Can't Buy Me Love—02 *	Drama	94578
2010-12-27	*Let's see the Meteor Shower together—24 *	Drama	146474
2010-12-28	*Triple Tap *	Film	111477
2010-12-29	*Triple Tap *	Film	111677
